# F197. PROMOTING MYELIN REPAIR RESCUES MICE FROM SCHIZOPHRENIA-LIKE BEHAVIOR INDUCED BY SOCIAL ISOLATION

**DOI:** 10.1093/schbul/sby017.728

**Published:** 2018-04-01

**Authors:** Lan Xiao

**Affiliations:** Third Military Medical University

## Abstract

**Background:**

Although pathological and genetic evidence suggest that oligodendrocyte (OL) or myelin deficits are associated with schizophrenia, the contribution of OL/myelin deficits to its etiology has not been clearly dissected, because OL/myelin abnormalities may be a concomitant phenomenon during the pathogenesis of schizophrenia.

**Methods:**

Using olig2 ablation specifically in OLs (olig2 CKO) mice, we detected myelin development status and animal behaviors under normal condition or subjected to social isolation. We also examined the therapeutic effect of FDA-approved compounds, like quetiapine (an APD) or clemastine (a histamine antagonist) on animal behaviors.

**Results:**

Our results demonstrated that deleting of olig2 leaded to impaired development of OLs and myelin deficit from postnatal day14 (P14) to P56, preferentially in cerebral cortex, and these young adult Olig2 KO mice showed anxiety-like behavior, motor skill learning deficit and cognitive deficit. Moreover, Olig2 CKO mice exhibited earlier social avoidance behavior than the WT littermates under prolonged social isolation, indicating that myelin deficit may enhance risk of schizophrenia upon environmental stress attacking. Interestingly, enhancing oligodendrocyte generation and myelin repair by quetiapine or clemastine successfully reversed the above phenotype.

**Discussion:**

Taking together, promoting myelin repair may present a new therapeutic strategy against schizophrenia.

